# The Complementary Asymmetric-Odds Weibull Distribution: A Flexible Lifetime Model with Applications to Hazard Rate Modeling and Change-Point Analysis

**DOI:** 10.3390/e28080891

**Published:** 2026-08-07

**Authors:** Dawlah Alsulami

**Affiliations:** Department of Statistics, Faculty of Science, King Abdulaziz University, Jeddah 21589, Saudi Arabia; dalsulami@kau.edu.sa

**Keywords:** change point, hazard rate function, lifetime data, methods of estimation, simulation, Weibull distribution

## Abstract

This paper introduces the Complementary Asymmetric-Odds Weibull (CAO–W) distribution, a new three-parameter distribution that improves the accuracy of modeling lifetime data with complex behavior. The proposed distribution is based on an asymmetric transformation that combines the baseline distribution with its complement, providing more flexibility when modeling a variety of hazard patterns, such as increasing, decreasing, and bathtub-shaped. Some statistical properties of the CAO–W distribution were studied including a mathematical and graphical analysis of the hazard rate function (HRF). The model parameters were estimated using four widely used estimation approaches: maximum likelihood (ML), least squares (LS), maximum product of spacings (MPS), and the Cramér-von-Mises (CVM). The efficiency of these approaches in estimating the model parameters was investigated through simulation studies under different scenarios. Moreover, the proposed distribution was applied to four real datasets, and compared to some flexible distributions to demonstrate its ability to provide a good fit for lifetime data in survival and reliability analysis applications. Finally, a change point analysis, based on the Minimum Information Criterion (MIC), is also conducted to highlight the flexibility of the proposed model.

## 1. Introduction

Accurately describing the behavior of the hazard rate function (HRF) has become a key challenge in analyzing lifetime data, as any limitation in representing this behavior will affect the predictive performance and inferential reliability. Classical distributions, including the Gompertz (Gomp) distribution [1], the Gamma distribution [2], and the Weibull (W) distribution [3] have been widely utilized to model failure patterns in lifetime data due to their simplicity and ability to capture basic hazard behaviors. In addition, the log-logistic (LL) distribution [4], although classical, offers greater flexibility in capturing unimodal hazard pattern and heavy-tailed data, making it particularly useful in practical applications. Nevertheless, in some instances, these distributions do not adequately capture complex hazard behavior, particularly nonlinear patterns.

This limitation has led to extensive work being conducted to generalize classical distributions in order to improve their flexibility. Initially, efforts were focused on improving the adaptability of the classical distributions by including an additional parameter to improve their ability to model real life data. One of the earliest examples is the generalized gamma (GG) distribution, introduced by [5], to model a wide range of lifetime applications. As another example, the exponentiated Weibull (EW) distribution, defined by [6], is proposed to represent complex lifetime data. Since then these distributions have frequently been used to accommodate various data behavior.

Furthermore, a considerable amount of work in the literature has developed systematic generator mechanisms whereby any baseline distribution can be extended by applying a functional transformation. Through this approach, various flexible families of distributions have been introduced. For example, the Marshall–Olkin family by [7], the beta-generated family by [8], the Kumaraswamy-generated family by [9], the transform transformer family of distributions by [10], the exponentiated generalized family by [11], and the odd-based family by [12]. While these families have provided greater flexibility for classical lifetime distributions, each transforms the baseline distribution in a different way, resulting in different distributional characteristics and hazard rate patterns. In addition, greater flexibility is often achieved by introducing additional shape parameters, which may increase model complexity and complicate parameter estimation and interpretation, whereas others rely on specific transformation strategies based on the cumulative distribution function (CDF), the survival function, or the odds ratio, each emphasizing different aspects of the baseline distribution. Consequently, there remains scope for exploring alternative transformation strategies that preserve a simple model structure while allowing greater flexibility in modeling hazard rate behavior. These approaches have inspired numerous authors to introduce more flexible families of distributions; see [13,14,15,16,17,18,19,20], among others.

Recently, flexible distributions capable of fitting complex hazard patterns, tail behavior, and early-life failures are in growing demand, as such data are often inadequately handled by classical models. Therefore, more effort has been devoted to developing new distributions that balance structural simplicity with flexibility. As a result, a variety of flexible models have been developed in the literature, such as, the flexible EW distribution proposed by [21], the Burr XII–Gompertz distribution introduced by [22], the new flexible odd type-G family of distributions suggested by [23], and the Kumaraswamy alpha-power log-logistic distribution by [24]. More recently, additional families have been proposed to further increase modeling flexibility through alternative transformation mechanisms, including the Cosine Kumaraswamy family [25] and the Logarithmic Cosine-G family [26].

In spite of these advances, some of the introduced distributions have complex mathematical structures, resulting in difficulties when estimating parameters or interpreting results. Other distributions, regardless of having multiple parameters, do not always offer enough flexibility to model various forms of the HRF that adequately reflect the observed data behavior. Motivated by these observations, a new complementary asymmetric-odds transformation is introduced by integrating the cumulative distribution function and its survival function through complementary exponents. Rather than being constructed solely from the odds ratio, the proposed transformation incorporates both components, allowing their relative contributions to be controlled by a single asymmetry parameter. This construction provides an alternative transformation mechanism that preserves structural simplicity while offering sufficient flexibility to represent diverse hazard rate patterns. Therefore, the Complementary Asymmetric-Odds Weibull (CAO–W) distribution is developed using the W distribution as the baseline model. The contributions of this paper can be summarized as follows:

- Develop a new extension of the W distribution with high flexibility in modeling various complex shapes of HRF.
- Demonstrate the effectiveness of the proposed distribution in capturing hazard patterns, including increasing, decreasing, and bathtub, within a single flexible model, which may not be adequately captured by classical distributions.
- Provide a practical interpretation of the lifetime data analysis by linking the model parameters to the shapes of the probability density function (PDF) and HRF.
- Evaluate the performance of four methods of estimation through simulation studies with different sample sizes.
- Show the improved fitting of the proposed distribution to real data relative to competing models in survival and reliability applications.
- Extend the applicability of the suggested distribution to analyze the structural changes in lifetime data using minimum information criterion (MIC)-based change-point method.

The remaining sections of the paper are outlined as follows. Section 2 introduces the motivation of the the proposed generator and construction of CAO–W distribution. Section 3 provides analytical and graphical analysis for the HRF. Some general mathematical properties of the proposed model are listed in Section 4, while parameter estimation using four selected approaches is presented in Section 5. In Section 6, three simulation studies with different sample sizes are shown. Moreover, the application and the change-point analysis for four datasets are given in Section 7. Finally, Section 8 summarizes the paper and highlights areas for future research.

## 2. Motivation and Interpretation of the Proposed Generator

Many existing generator families are developed by transforming the CDF, the survival function, or the odds ratio formed from these functions. However, the complementary asymmetric-odds transformation follows a different strategy by combining the cumulative distribution and survival functions through complementary exponents. Allowing their relative contributions to vary provides additional flexibility in controlling the shape of the resulting distribution. Accordingly, the proposed transformation provides a simple and interpretable framework for generating flexible lifetime distributions while preserving a parsimonious model structure. Using the complementary exponents *a* and 1−a, the complementary asymmetric-odds transformation is defined as follows:

(1) $$A\left(x\right)=\frac{G{\left(x\right)}^{a}}{{\left(1-G\left(x\right)\right)}^{1-a}}\;0<a<1.$$

The complementary exponents *a* and 1−a allow the balance between the cumulative distribution and survival functions to be adjusted through a single parameter. Therefore, the resulting distribution is capable of accommodating a wider range of hazard rate patterns while maintaining a simple model structure.

Taking logarithms results in

$$\log A\left(x\right)=a\log G\left(x\right)+\left(1-a\right)\log\frac{1}{1-G(x)},$$

indicating that parameter *a* can be considered as a weight between two components related to the lower and upper tails. Accordingly, small values of *a* emphasizes the upper-tail component, while large values highlight the lower-tail contribution. The intermediate scenario (a=0.5) reflects an even balance between the two.

### 2.1. Relationship with Existing Families

Although the proposed construction is conceptually related to several generated families, it is not a simple reparameterization of any of them. In particular,

$$A\left(x\right)=\frac{G{\left(x\right)}^{a}}{{\left(1-G\left(x\right)\right)}^{1-a}}={\left(\frac{G(x)}{1-G(x)}\right)}^{a}{\left(1-G\left(x\right)\right)}^{2a-1}.$$

The above decomposition shows that the proposed generator consists of an odd-type component together with an additional correction factor ${\left(1-G\left(x\right)\right)}^{2a-1}$. Except for the special case a=0.5, this correction term cannot be absorbed into the classical odd-G generator, indicating that the proposed construction is structurally different from the standard odd-G family. Table 1 presents a summary of the primary distinctions between the CAO–G family and other related families.

As shown in Table 1, unlike the Marshall–Olkin-G designs that modify only the survival function, the proposed generator simultaneously incorporates both the cumulative distribution and survival functions. Moreover, unlike the Beta–G and Kumaraswamy–G families, which achieve flexibility through transformations of the CDF, the proposed transformation is formulated directly through a cumulative hazard representation. Overall, the proposed generator differs from existing families not only in its mathematical formulation but also in the way flexibility is introduced. In particular, the complementary asymmetric transformation introduces a single asymmetry parameter that regulates the relative contributions of the cumulative distribution and survival functions, providing an alternative mechanism for generating diverse hazard rate patterns within a parsimonious model structure.

### 2.2. The Construction of the CAO–W Distribution

Let *X* be a random variable from a W distribution with shape parameter *c* and scale parameter β, then the CDF and the PDF, are respectively, written as

(2) $$\begin{array}{cc} G(x) & =1-\exp\;\left[-{\left(\frac{x}{\beta }\right)}^{c}\right],\;x>0,\;c>0,\;\beta >0, \end{array}$$

(3) $$\begin{array}{cc} g(x) & =\frac{c}{\beta }{\left(\frac{x}{\beta }\right)}^{c-1}\exp\;\left[-{\left(\frac{x}{\beta }\right)}^{c}\right]. \end{array}$$

Then, the proposed CAO–W distribution is constructed by applying the transformation in Equation (1) within the cumulative hazard formulation of the W distribution. Therefore, the CDF of the CAO–W takes the following form

(4) F(x)=1−exp{−A(x)},x>0.

Substituting Equation (1) into Equation (4), the CDF is given by

(5) $$F\left(x\right)=1-\exp\left\{-\exp\;\left[-\left(a-1\right){\left(\frac{x}{\beta }\right)}^{c}\right]{\left(1-\exp\;\left[-{\left(\frac{x}{\beta }\right)}^{c}\right]\right)}^{a}\right\},$$

and the corresponding PDF and HRF are, respectively, written as

(6) $$\begin{array}{cc} f(x)= & \frac{c}{\beta }{\left(\frac{x}{\beta }\right)}^{c-1}\exp\;\left[-\left(a-1\right){\left(\frac{x}{\beta }\right)}^{c}\right]{\left(1-\exp\;\left[-{\left(\frac{x}{\beta }\right)}^{c}\right]\right)}^{a-1}\\ \; & \;\times \left[\left(1-a\right)+\left(2a-1\right)\exp\;\left[-{\left(\frac{x}{\beta }\right)}^{c}\right]\right]\\ \; & \;\times \exp\left\{-\exp\;\left[-\left(a-1\right){\left(\frac{x}{\beta }\right)}^{c}\right]{\left(1-\exp\;\left[-{\left(\frac{x}{\beta }\right)}^{c}\right]\right)}^{a}\right\}, \end{array}$$

(7) $$\begin{array}{cc} h(x)= & \frac{c}{\beta }{\left(\frac{x}{\beta }\right)}^{c-1}\exp\;\left[-\left(a-1\right){\left(\frac{x}{\beta }\right)}^{c}\right]{\left(1-\exp\;\left[-{\left(\frac{x}{\beta }\right)}^{c}\right]\right)}^{a-1}\\ \; & \;\times \left[\left(1-a\right)+\left(2a-1\right)\exp\;\left[-{\left(\frac{x}{\beta }\right)}^{c}\right]\right]. \end{array}$$

**Figure 1:**
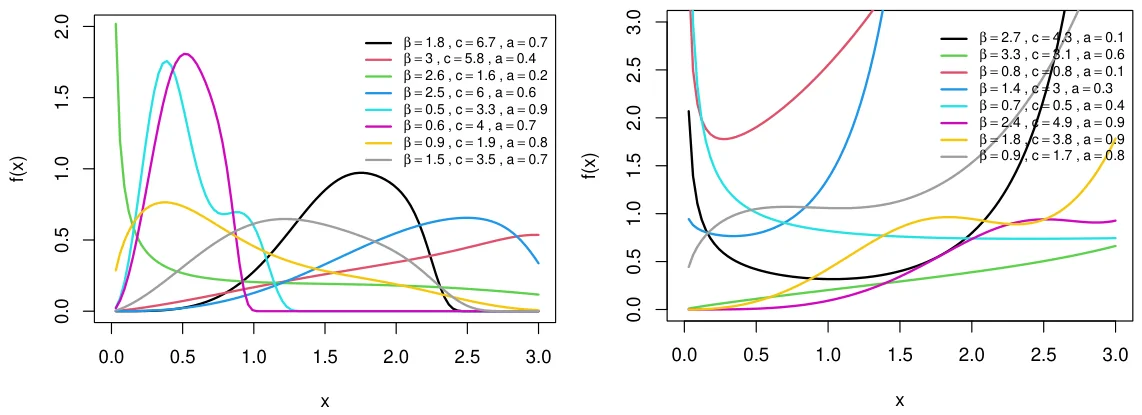
Plots of the PDF (**right**) and HRF (**left**) for different parameter values.

Figure 1 presents different shapes of the PDF and HRF of the CAO-W distribution under various scenarios.

Figure 1 illustrates various shapes of the PDF and HRF of the proposed CAO–W distribution under various parameter combinations. As shown, the shapes of both functions vary considerably across different combinations of the model parameters. In particular, for the parameter combinations considered, the PDF ranges from monotone decreasing to unimodal forms with different degrees of skewness and dispersion, whereas the HRF exhibits increasing, decreasing, bathtub-shaped, and unimodal behaviors. These graphical results are consistent with the analytical findings and visually highlight the diverse density and hazard shapes accommodated by the proposed model.

## 3. Hazard Rate Analysis

The HRF plays a critical role as a tool for analyzing lifetime distributions. In this section, the behavior of the HRF is examined to investigate the influence of the model parameters in modeling various hazard patterns.

### 3.1. Behavior at Origin

As x→0, then ${\left(\frac{x}{\beta }\right)}^{c}\to 0.$ Therefore, using the following approximation

$$1-e^{\left(-z\right)}∼z,\;\;\;as\;z\to 0,$$

leads to

$${\left(1-\exp\;\left[-{\left(\frac{x}{\beta }\right)}^{c}\right]\right)}^{a-1}∼{\left(\frac{x}{\beta }\right)}^{c(a-1)}.$$

Moreover,

$$\left(1-a\right)+\left(2a-1\right)\exp\;\left[-{\left(\frac{x}{\beta }\right)}^{c}\right]\to a.$$

Thus, as x→0,

$$h\left(x\right)∼\frac{ac}{\beta }{\left(\frac{x}{\beta }\right)}^{ca-1}.$$

Hence, the hazard behavior near the origin is governed by the product ca as follows:

$$\lim_{x\to 0}h\left(x\right)=\left\{\begin{array}{cc} \infty , & ca<1,\\ \frac{1}{\beta }, & ca=1,\\ 0, & ca>1. \end{array}\right.$$

These results indicate that when ca<1, the hazard starts at a high level, whereas for ca>1, it begins from zero.

### 3.2. Behavior as x→∞

As x→∞, then ${\left(\frac{x}{\beta }\right)}^{c}\to \infty$. Moreover,

$$\exp\;\left[-{\left(\frac{x}{\beta }\right)}^{c}\right]\to 0\;\mathrm{and}\;1-\exp\;\left[-{\left(\frac{x}{\beta }\right)}^{c}\right]\to 1.$$

Also,

$$\left(1-a\right)+\left(2a-1\right)\exp\;\left[-{\left(\frac{x}{\beta }\right)}^{c}\right]\to 1-a.$$

Therefore, as x→∞,

$$h\left(x\right)∼\frac{c(1-a)}{\beta }{\left(\frac{x}{\beta }\right)}^{c-1}\exp\;\left\{\left(1-a\right){\left(\frac{x}{\beta }\right)}^{c}\right\}.$$

Since 1−a>0, the last term tends to *∞*, and dominates the second factor ${\left(\frac{x}{\beta }\right)}^{c-1}$. Then

h(x)→∞,asx→∞.

It is easily seen that, in the right tail of the distribution, the HRF eventually increases without bound. This indicates that, for 0<a<1, the HRF is unbounded in the right tail. The aforementioned unbounded right-tail behavior may not be suitable for many applications. Nonetheless, this characteristic aligns with systems exhibiting significant aging or progressive decline, wherein the probability of failure raises continually over time. Consequently, the suggested model is especially appropriate for lifespan data with substantial wear-out features. Therefore, The CAO–W distribution parameters play distinct roles in determining the shape of the HRF. In particular, the parameter *a* contributes to the increase of the HRF for large *x*, affecting the tail behavior. Furthermore, the parameter *c* determines the rate of change of the HRF, whereas the scale parameter β determines where the HRF is shifted along the *x*-axis. Therefore, the asymptotic findings demonstrate the impact of the parameters on the behavior of the HRF at the origin and in the right tail. The product ca explains the initial behavior of the hazard, while the parameter *a* regulates the tail behavior.

### 3.3. Hazard Rate Flexibility

**Figure 2:**
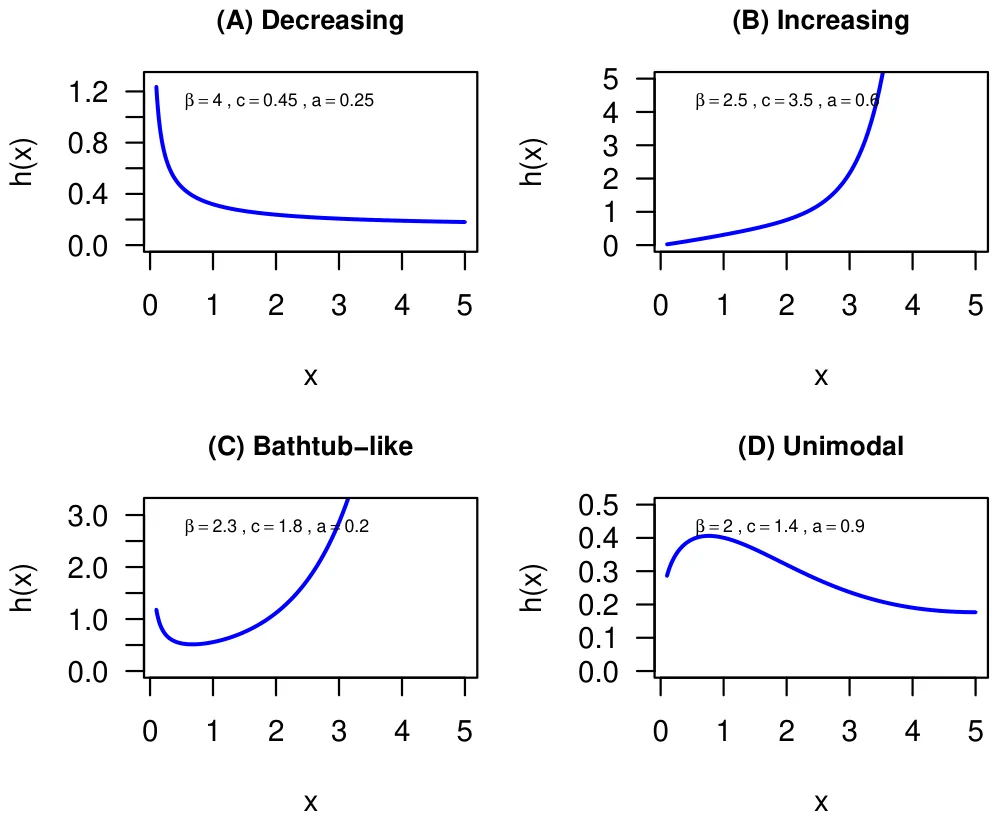
Plot of HRF of the CAO–W for some values of parameters.

The interactions between the model parameters provide the CAO–W model with the ability to generate a variety of hazard rate patterns. The introduction of the parameter *a* enables the cumulative hazard generator to incorporate varying contributions from the lower and upper ends, in contrast to the classical W distribution, which is limited to monotone behaviors. Therefore, it is possible to acquire additional flexibility without a significant increase in the number of parameters. The proposed CAO–W distribution has the ability to accommodate a wide range of important failure rate shapes as shown in Figure 2.

In particular, early-life failures are linked to decreasing hazards whereas ageing or wear-out mechanisms are marked by increasing hazards. Bathtub-shaped hazards represent systems with an infant mortality and wear-out effects, whereas unimodal hazards represent the situations in which the risk initially increases to a peak and subsequently declines. Hence, the proposed distribution provides a flexible alternative for modeling data in different reliability and survival applications. It is worth mentioning that the suggested model does not claim the exclusiveness of generating bathtub or non-monotone HRFs since such behaviors have been seen for many existing lifespan distributions. Rather, the complementary asymmetric cumulative-hazard structure offers an interpretable alternative mechanism by which a diverse set of hazard patterns can be acquired in a relatively simple parametric framework.

## 4. Mathematical Properties

### 4.1. Quantile Function

The quantile function of the CAO–W distribution is given by setting F(x)=u, where u∈(0,1), as follows

$$1-\exp\left\{-\exp\;\left[-\left(a-1\right){\left(\frac{x}{\beta }\right)}^{c}\right]{\left(1-\exp\;\left[-{\left(\frac{x}{\beta }\right)}^{c}\right]\right)}^{a}\right\}=u.$$

Therefore,

(8) $$\exp\;\left[-\left(a-1\right){\left(\frac{x}{\beta }\right)}^{c}\right]{\left(1-\exp\;\left[-{\left(\frac{x}{\beta }\right)}^{c}\right]\right)}^{a}=-\ln\left(1-u\right).$$

Now, let

(9) $$y=\exp\;\left[-{\left(\frac{x}{\beta }\right)}^{c}\right].$$

Then Equation (8) reduces to

(10) $$\frac{{\left(1-y\right)}^{a}}{y^{1-a}}=-\ln\left(1-u\right).$$

In spite of the lack of a closed-form solution for the above equation, numerical methods can be employed to obtain the solution. Therefore, after calculating the solution $y_{u}\in \left(0,1\right)$ numerically and from Equation (9), the quantile is computed directly as follows

$$x=\beta {\left(-\ln y_{u}\right)}^{1/c}.$$

It is worth highlighting that, since the left hand side of Equation (10) is continuous and strictly decreasing, while the right hand side is always positive for $y_{u}\in \left(0,1\right)$, the quantile function exists and is unique (Algorithm 1).
**Algorithm 1** Generation of random observations from the CAO–W distribution.**Input:** Parameters β>0, c>0, 0<a<1, and sample size *n*.1.For i=1,…,n, generate a random number $U_{i}∼U\left(0,1\right)$.2.
Compute $w_{i}=-\ln\left(1-U_{i}\right)$.
3.Determine the unique solution $y_{i}\in \left(0,1\right)$ of ${\left(1-y_{i}\right)}^{a}-w_{i}y_{i}^{1-a}=0$ using a numerical root-finding procedure. In this study, Brent’s method was employed through the uniroot() routine in R version 4.5.3.4.Obtain the corresponding CAO–W random variate$$X_{i}=\beta {\left(-\ln y_{i}\right)}^{1/c}.$$5.Repeat Steps 1–4 until *n* observations are generated.**Output:** A random sample $X_{1},X_{2},\ldots ,X_{n}$ from the CAO–W distribution.

### 4.2. Moments

Moments are fundamental in statistical distributions They describe the main features of a probability distribution, including its location, variability, asymmetry, and tail behavior. The moments of the CAO–W distribution is described in the following theorem.

**Theorem** **1.** *Let X∼CAO-W(β,c,a) with β>0, c>0, and 0<a<1. Then the rth moment of the CAO–W takes the following form:*

(11) μr′=∑j=0∞∑i=0∞(−1)j+ij!a(j+1)−1iA1+A2,

*where*

(12) $$\begin{array}{cc} A_{1} & =\left(1-a\right)\int_{0}^{\infty }x^{r}\frac{c}{\beta }{\left(\frac{x}{\beta }\right)}^{c-1}\exp\;\left[-\left((a-1)(j+1)+i\right){\left(\frac{x}{\beta }\right)}^{c}\right]dx, \end{array}$$

(13) $$\begin{array}{cc} A_{2} & =\left(2a-1\right)\int_{0}^{\infty }x^{r}\frac{c}{\beta }{\left(\frac{x}{\beta }\right)}^{c-1}\exp\;\left[-\left((a-1)(j+1)+i+1\right){\left(\frac{x}{\beta }\right)}^{c}\right]dx. \end{array}$$

**Proof.**  For a continuous random variable *X*, the *rth* moment $\mu_{r}^{\prime }=E\left(X^{r}\right)$ is given by

$$\mu_{r}^{\prime }=E\left(X^{r}\right)=\int_{0}^{\infty }x^{r}f\left(x\right)\;dx.$$

Thus, for X∼CAO-W(β,c,a),

$$\begin{array}{cc} \mu_{r}^{\prime }\; & =\int_{0}^{\infty }x^{r}\frac{c}{\beta }{\left(\frac{x}{\beta }\right)}^{c-1}\exp\;\left[-\left(a-1\right){\left(\frac{x}{\beta }\right)}^{c}\right]{\left(1-\exp\;\left[-{\left(\frac{x}{\beta }\right)}^{c}\right]\right)}^{a-1}\\ \; & \;\times \left[\left(1-a\right)+\left(2a-1\right)\exp\;\left[-{\left(\frac{x}{\beta }\right)}^{c}\right]\right]\\ \; & \;\times \exp\;\left\{-\exp\;\left[-\left(a-1\right){\left(\frac{x}{\beta }\right)}^{c}\right]{\left(1-\exp\;\left[-{\left(\frac{x}{\beta }\right)}^{c}\right]\right)}^{a}\right\}\;dx. \end{array}$$

Using the following series expansion on the last exponential term

(14) $$e^{-y}=\sum_{j=0}^{\infty }\frac{{\left(-1\right)}^{j}}{j!}y^{j},$$

we get

$$\begin{array}{cc} \mu_{r}^{\prime }\; & =\sum_{j=0}^{\infty }\frac{{\left(-1\right)}^{j}}{j!}\int_{0}^{\infty }x^{r}\frac{c}{\beta }{\left(\frac{x}{\beta }\right)}^{c-1}\exp\;\left[-\left(a-1\right)\left(j+1\right){\left(\frac{x}{\beta }\right)}^{c}\right]\\ \; & \;\times {\left(1-\exp\;\left[-{\left(\frac{x}{\beta }\right)}^{c}\right]\right)}^{a(j+1)-1}\left[\left(1-a\right)+\left(2a-1\right)\exp\;\left[-{\left(\frac{x}{\beta }\right)}^{c}\right]\right]dx. \end{array}$$

Now, applying the following generalized binomial expansion

(15) (1−y)ν=∑i=0∞(−1)iνkyi.

The *rth* moment is expressed as follows:

μr′=∑j=0∞∑i=0∞(−1)j+ij!a(j+1)−1i∫0∞xrcβxβc−1×exp−i+(j+1)(a−1)xβc(1−a)+(2a−1)exp−xβcdx,=∑j=0∞∑i=0∞(−1)j+ij!a(j+1)−1iA1+A2,

where $A_{1}$ and $A_{2}$ are defined in the theorem. Therefore, the *rth* moment is obtained by calculating the quantities $A_{1}$ and $A_{2}$ numerically. □

#### Moment-Based Measures

To further describe the CAO–W distribution, some measures based on moments are also considered.

Mean and Variance

To get a basic insight into the location and dispersion of the CAO–W distribution, the mean and variance can be used. These two measures can be calculated directly from Equation (11) as follows:

$$\begin{array}{cc} \mathrm{Mean} & =\mu_{1}^{\prime },\\ \mathrm{Variance} & =\mu_{2}^{\prime }-{\left(\mu_{1}^{\prime }\right)}^{2}. \end{array}$$

Coefficient of Variation

As a measure of variability relative to the mean, the coefficient of variation (CV) produces a more accurate comparison of dispersion across parameter cases. The CV is given by the following formula

$$\begin{array}{cc} \mathrm{CV} & =\frac{\sqrt{\mathrm{Variance}}}{\mathrm{Mean}}. \end{array}$$

Skewness and Kurtosis

In statistics, skewness (SK) and kurtosis (K) are important measures of the distribution’s shape. The SK gives insight into the degree of asymmetry, while the K offers information about tail behavior. These two measures are, respectively, given by

$$\begin{array}{cc} SK & =\frac{{\mu_{3}}^{\prime }-3{\mu_{2}}^{\prime }{\mu_{1}}^{\prime }+2{\mu_{1}^{\prime }}^{3}}{{\left({\mu_{2}}^{\prime }-{\mu_{1}^{\prime }}^{2}\right)}^{\frac{3}{2}}},\\ K & =\frac{\mu_{4}^{\prime }-4\mu_{3}^{\prime }\mu_{1}^{\prime }+6\mu_{2}^{\prime }{\mu_{1}^{\prime }}^{2}-3{\mu_{1}^{\prime }}^{4}}{{\left(\mu_{2}^{\prime }-{\mu_{1}^{\prime }}^{2}\right)}^{2}}. \end{array}$$

Statistics software such as R can be used to calculate the measures outlined above. Accordingly, Table 2 identifies the values of CV, SK, and K for some cases of parameters that increase together, rather than focusing on the effect of each parameter individually.

**Figure 3:**
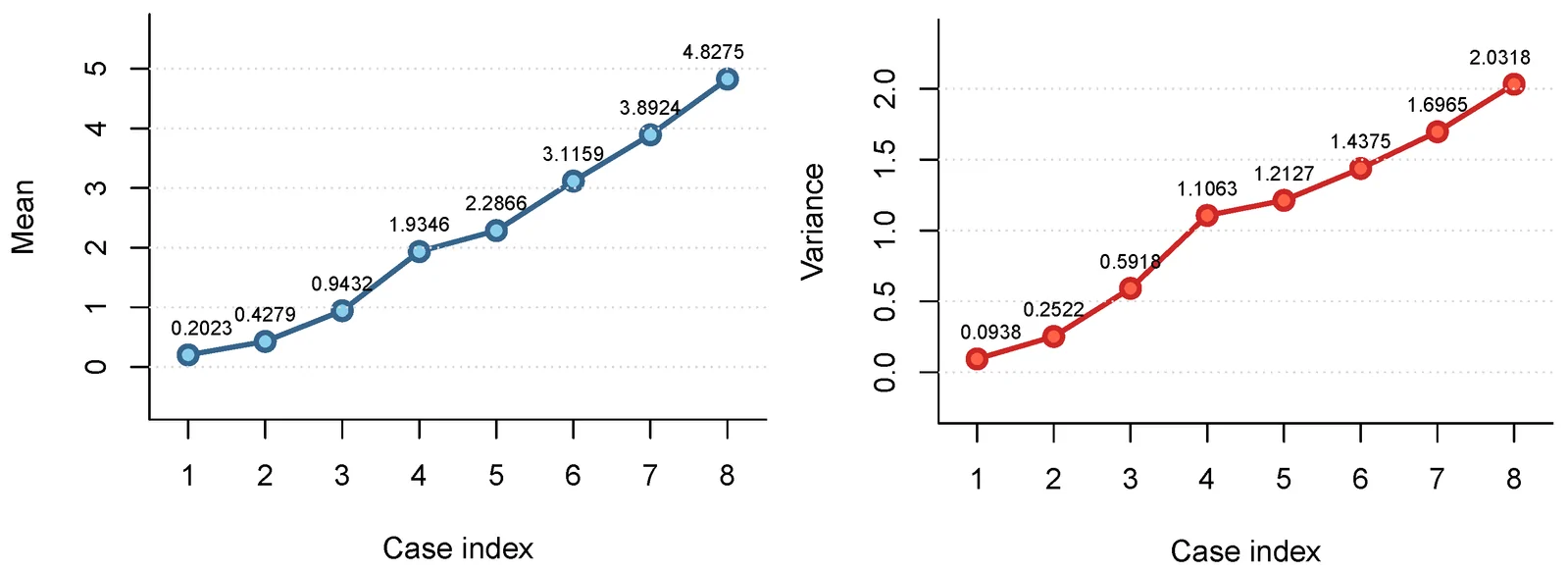
Plot of the mean (**left**) and variance (**right**) for the selected parameters.

Figure 3 presents the mean and variance for the selected parameters, while Table 2 lists the values of CV, SK, and K.

Form Figure 3, it is evident that the mean and variance increase with a rise in the parameter values. Moreover, it can be seen from Table 2 that there is a clear relationship between the dispersion of the distribution and its overall shape. It is clear from the CV that as parameter values decrease, the observations become more dispersed. In contrast, when the parameters increase, the CV decreases, resulting in more condensed observations. In parallel, the changes in SK and K indicate a shift from a right-skewed and heavy-tailed (Leptokurtic) distribution to a more symmetric and eventually left-skewed shape with lighter tails (Platykurtic). Therefore, the CAO–W distribution provides flexibility to capture a wide range of data behaviors. Moreover, the effect of each model parameter was explored by varying one parameter at a time while holding the other two constant at the representative baseline values (β,c,a)=(2.5,3.1,0.4). Table 3 summarizes the influence of each parameter on the descriptive measures of the CAO–W distribution.

It is shown from Table 3 that when β increases, both the mean and variance increase, whereas the SK, K, and CV remain constant, indicating that β primarily acts as a scale parameter. On the other hand, increasing parameter *c* is associated with a decrease in the variance, SK, and CV, showing the important role of *c* in controlling the variability and shape of the distribution. In addition, the parameter *a* has a clear effect on the mean, variance, SK, K, and CV, giving the model greater flexibility to represent different distributional shapes.

### 4.3. Moment Generating Function

The moment generating function of the random variable *X* from the CAO–W distribution is expressed as

$$M_{X}\left(t\right)=E\left(e^{tX}\right)=\int_{0}^{\infty }e^{tx}f\left(x\right)\;dx.$$

Using the series Equation (14) and the generalized binomial expansion (15), the moment generating function takes the following form

MX(t)=∑j=0∞∑i=0∞(−1)j+ij!a(j+1)−1i∫0∞etxcβxβc−1×exp−k−(j+1)(1−a)xβc(1−a)+(2a−1)exp−xβcdx.

Now, using the power series expansion

$$e^{tx}=\sum_{k=0}^{\infty }\frac{t^{k}}{k!}\;x^{k},$$

the moment generating function is given by

MX(t)=∑j=0∞∑i=0∞∑k=0∞(−1)j+itkj!k!a(j+1)−1i∫0∞xkcβxβc−1×exp−k−(j+1)(1−a)xβc(1−a)+(2a−1)exp−xβcdx.

Therefore, similar to the moments, the moment generating function is obtained numerically from the above representation.

### 4.4. Rényi Entropy

The Rényi entropy is introduced by [28] as a flexible measure of uncertainty depending on the tuning parameter δ. The Rényi entropy is given by

(16) $$R_{\delta }\left(x\right)=\frac{1}{1-\delta }\log\left(\int_{0}^{\infty }f^{\delta }\left(x\right)\;dx\right),$$

where δ>0 and δ≠1. Therefore,

$$\begin{array}{cc} \int_{0}^{\infty }f^{\delta }\left(x\right)\;dx & ={\left(\frac{c}{\beta }\right)}^{\delta }\int_{0}^{\infty }{\left(\frac{x}{\beta }\right)}^{\delta (c-1)}\exp\;\left[-\delta \left(a-1\right){\left(\frac{x}{\beta }\right)}^{c}\right]{\left(1-\exp\;\left[-{\left(\frac{x}{\beta }\right)}^{c}\right]\right)}^{\delta (a-1)}\\ \; & \;\times {\left[\left(1-a\right)+\left(2a-1\right)\exp\;\left[-{\left(\frac{x}{\beta }\right)}^{c}\right]\right]}^{\delta }\\ \; & \;\times \exp\;\left\{-\delta \exp\;\left[-\left(a-1\right){\left(\frac{x}{\beta }\right)}^{c}\right]{\left(1-\exp\;\left[-{\left(\frac{x}{\beta }\right)}^{c}\right]\right)}^{a}\right\}\;dx. \end{array}$$

Applying Equations (14) and (15), we get

∫0∞fδ(x)dx=cβδ∑i=0∞∑j=0∞(−1)i+jδii!ia+δ(a−1)j∫0∞xβδ(c−1)×exp−(δ+i)(a−1)+jxβc(1−a)+(2a−1)exp−xβcδdx.

Hence, the Rényi entropy is given by

(17) Rδ(x)=11−δlog{cβδ∑i=0∞∑j=0∞(−1)i+jδii!ia+δ(a−1)j∫0∞xβδ(c−1)×exp−(δ+i)(a−1)+jxβc(1−a)+(2a−1)exp−xβcδdx}.

Now if $|\frac{2a-1}{1-a}<1|$, then

$$\left|\frac{\left(2a-1\right)e^{-{\left(\frac{x}{\beta }\right)}^{c}}}{1-a}\right|<1,$$

and hence the following generalized binomial expansion can be applied to the last term in Equation (17).

(x+y)b=∑m=0∞bmxb−mym.

Thus, the Rényi entropy is simplified to

Rδ(x)=11−δlog{cβδ∑i=0∞∑j=0∞∑m=0∞(−1)i+jδii!ia+δ(a−1)jδm(1−a)δ−m(2a−1)m×∫0∞xβδ(c−1)exp−(δ+i)(a−1)+j+mxβcdx}

Therefore, the above representation of the Rényi entropy can be evaluated numerically.

### 4.5. Order Statistics

In reliability and survival analysis, order statistics provide information regarding the ordered observations as well as the extreme ones, such as minimums and maximums. Let $X_{1},\;X_{2},...,X_{n}$ be a random sample from the CAO–W, the PDF of the $i^{th}$ order statistics $X_{i:n}$ is expressed as follows

$$f_{i:n}\left(x\right)=\frac{n!}{(i-1)!(n-i)!}\;f\left(x\right)\;{\left[F\left(x\right)\right]}^{i-1}{\left[1-F\left(x\right)\right]}^{n-i}.$$

Using binomial expansion (15),

(18) fi:n(x)=n!(i−1)!(n−i)!∑s=0n−i(−1)sn−isf(x)[F(x)]s+i−1.

By substituting Equations (5) and (6) in Equation (18) and applying the binomial expansion (15) and the power expansion (14), the PDF of the *ith* order statistics is simplified to

fi:n(x)=∑r=0∞∑m=0∞∑k=0∞∑s=0n−in!(i−1)!(n−i)!(−1)s+k+m+rm!k+1mn−iss+i−1k×a(m+1)−1rcβcxc−1exp−(m+1)(a−1)+rxβc×(1−a)+(2a−1)exp−xβc.

## 5. Parameter Estimation

In order to investigate their efficiency and accuracy of the parameters’ estimates, four commonly used methods of estimation are used. These methods are chosen due to their widespread use in the literature and their ability to handle flexible models. These methods are briefly explained below.

### 5.1. Maximum Likelihood

The maximum likelihood (ML) is one of widely applied technique for estimating model parameters due to its flexibility, efficiency, and ability to produce consistent estimates. In the ML method, the estimates of the model parameters are determined by maximizing the log-likelihood function. Let $x_{1},x_{2},\ldots ,x_{n}$ be a sample from the CAO—W distribution, the log-likelihood function is written as

(19) $$\begin{array}{cc} \ell (\beta ,c,a)\; & =n\log c-n\log\beta +\left(c-1\right)\sum_{i=1}^{n}\log\left(\frac{x_{i}}{\beta }\right)\\ \; & \;-\left(a-1\right)\sum_{i=1}^{n}{\left(\frac{x_{i}}{\beta }\right)}^{c}+\left(a-1\right)\sum_{i=1}^{n}\log\left(1-\exp\left(-{\left(\frac{x_{i}}{\beta }\right)}^{c}\right)\right)\\ \; & \;+\sum_{i=1}^{n}\log\left[\left(1-a\right)+\left(2a-1\right)\exp\left(-{\left(\frac{x_{i}}{\beta }\right)}^{c}\right)\right]\\ \; & \;-\sum_{i=1}^{n}\exp\left(-\left(a-1\right){\left(\frac{x_{i}}{\beta }\right)}^{c}\right){\left(1-\exp\left(-{\left(\frac{x_{i}}{\beta }\right)}^{c}\right)\right)}^{a}. \end{array}$$

Maximizing Equation (19) numerically using any optimization function such as optim() in the statistical software R version 4.5.3 results in the estimates of the parameters. As an alternative, the ML estimates can be obtained by differentiating Equation (19) with respect to each parameter and equating the resulting equations to zero and solve them analytically. It is important to note that the likelihood function of the proposed model is highly nonlinear, making a general analytical proof of the global uniqueness of the ML estimate challenging. Although the estimation procedure achieved consistently high convergence rates throughout the simulation study, these empirical results should not be interpreted as a theoretical proof of the uniqueness of the ML. A rigorous theoretical investigation of this property is left for future research.

### 5.2. Least Squares

The least squares (LS) method is one of the simplest estimation methods that is commonly used in the statistical literature due to its simplicity and ease of implementation. It is considered useful alternative to the ML because of its robustness and straightforward computational procedure. The estimates are obtained by minimizing the squared differences between the empirical and theoretical distribution functions. Let $x_{\left(1\right)},x_{\left(2\right)},\ldots ,x_{\left(n\right)}$ be the ordered sample from the CAO—W distribution, the LS method minimizes the following objective function

(20) $$LS\left(\beta ,c,a\right)=\sum_{i=1}^{n}{\left[F\left[x_{\left(i\right)}\right]-\frac{i}{n+1}\right]}^{2},$$

where $F[x_{\left(i\right)}]$ is the CDF of the CAO–W distribution as defined in Equation (5). Minimizing Equation (20), using, for example, *optim* function in R, or alternatively obtaining the derivative of (20) with respect to β,c, and *a*, setting them to zero, and solve them produces the LS estimates of the model parameters.

### 5.3. Maximum Product of Spacings

The maximum product of the spacings (MPS) method has attracted the attention in the literature as it is numerically stable and performs well, especially in small samples. The idea of the MPS method is to maximize the spacings between consecutive values of the distribution function. Let $x_{\left(1\right)},x_{\left(2\right)},\ldots ,x_{\left(n\right)}$ be the ordered sample from the CAO–W distribution, the MPS method maximizing the following objective function

(21) $$MPS\left(\beta ,c,a\right)=\frac{1}{n+1}\sum_{i=1}^{n+1}\log D_{i},$$

where $D_{i}=F\left[x_{\left(i\right)}\right]-F\left[x_{\left(i-1\right)}\right],\;i=1,2,\ldots ,n+1$ is the uniform spacings between consecutive ordered values. Similarly, maximizing Equation (21) using the *optim* function, or equivalently, setting the first derivative of (21) with respect to each parameter to zero and solve the resulting equations, provides the estimates of the MPS technique.

### 5.4. Cramér–Von Mises

The idea of Cramér–von Mises (CVM) method is to minimize the goodness-of-fit between the empirical distribution function and its corresponding theoretical one. It attracts attention in the literature due to its sensitivity to extreme observations, and is therefore considered a useful estimation method for complex models. Let $x_{\left(1\right)},x_{\left(2\right)},\ldots ,x_{\left(n\right)}$ be the ordered sample drawn from the CAO–W distribution, the CVM method minimizes the following function

(22) $$CVM\left(\beta ,c,a\right)=\frac{1}{12n}+\sum_{i=1}^{n}{\left[\left[F(x_{\left(i\right)})\right]-\frac{2i-1}{2n}\right]}^{2}.$$

The CVM estimates are obtained by minimizing Equation (22) numerically using, for example, the optim () function. Another option to obtain the CVM estimates is to equalize the first derivative of (22) with respect to β,c and *a* to zero and solve these equations analytically.

## 6. Simulation Study

This section focuses on evaluating and comparing the performance of the four methods of estimation: ML, LS, MPS, and CVM in estimating the distribution parameters through simulation studies. Different sample sizes n=25,50,100,200,400 are generated with the number of simulations $N_{sim}=5000$. The following three cases of parameters are utilized.

Case1:β=1.8,c=4.7,a=0.8;

Case2:β=1.2,c=1.5,a=0.6;

Case3:β=2.5,c=3.1,a=0.4.

**Figure 4:**
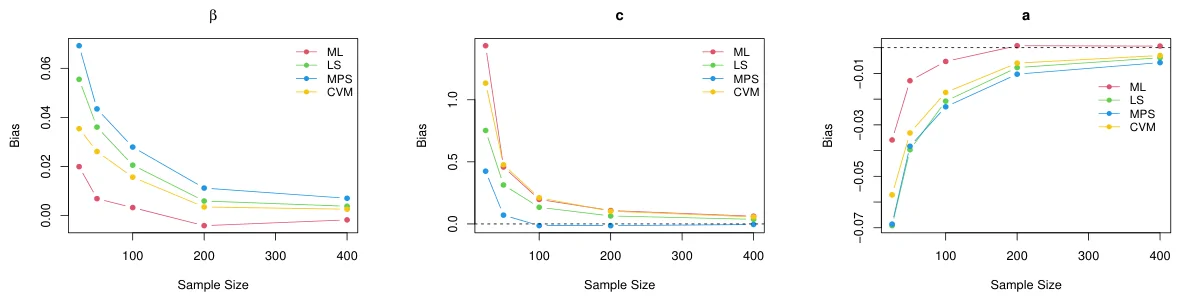
Plot of the bias of the estimates for the CAO-W distribution for Case I.

**Figure 5:**
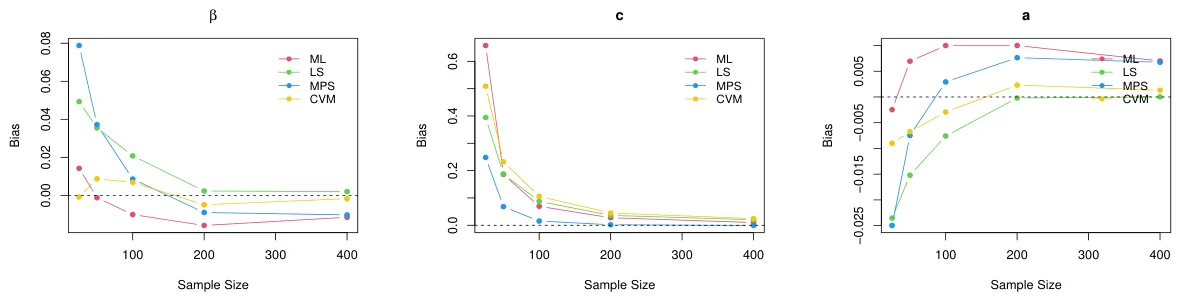
Plot of the bias of the estimates for the CAO-W distribution for Case II.

**Figure 6:**
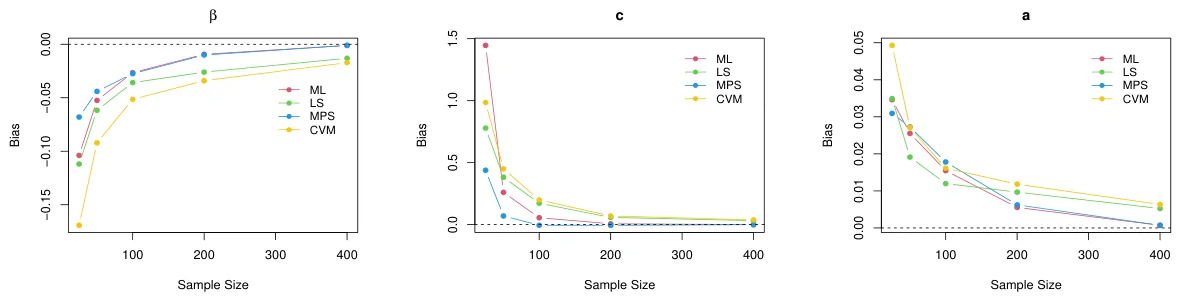
Plot of the bias of the estimates for the CAO-W distribution for Case III.

The root mean squared error (RMSE), the convergence rate (CR), and the bias are used to assess the performance of estimates. These measures are, respectively, calculated by

$$RMSE\left(\hat{\Lambda }\right)=\sqrt{\frac{1}{N_{sim}}\sum_{v=1}^{N_{sim}}{\left({\hat{\Lambda }}_{v}-\Lambda \right)}^{2}},$$

$$\mathrm{CR}\left(\%\right)=\frac{100}{N_{sim}}\sum_{i=1}^{N_{sim}}conv_{v},$$

where $conv_{v}=1$ if the optimization procedure converges, and the resulting estimates fulfill the parameter constraints, and 0 otherwise.

$$bias\left(\hat{\Lambda }\right)=\frac{1}{N_{sim}}\sum_{v=1}^{N_{sim}}\left({\hat{\Lambda }}_{v}-\Lambda \right),$$

where Λ=(β,c,a) represent the true values of parameters, and $\hat{\Lambda }$ represent their estimates. Table 4 lists the values of the estimates, their RMSE, and the CR, whereas Figure 4, Figure 5 and Figure 6 show the bias for different estimation methods.

It is evident from Figure 4, Figure 5 and Figure 6 that overall, the estimated bias generally approached zero as the sample size increased, although this trend was not strictly monotonic for all parameters. The slight increases observed for parameter *a* in Cases I and II and parameter β in Case III occurred because the signed bias became less negative as the sample size increased, while still approaching zero overall. From Table 4, the first notable finding is that all methods of estimation exhibited the consistency property as the estimators approach their true values when the sample size increases. As a result, the RMSE values decreases with an increase in the sample size, indicating a gradual improvement in the estimation accuracy. Furthermore, all estimation techniques achieved a high convergence rate, exceeding 99.7% for the smallest sample size and approaching 100% for moderate and large sample sizes, confirming the numerical stability of the estimation algorithms. For case I, the MPS method produced the smallest RMSE values for the shape parameter *c* across most sample sizes. However, the differences between all methods were relatively small for the parameters β and *a*. The ML method was less stable in estimating the shape parameter *c* when n=25 in case II, producing a noticeably larger RMSE than the other methods. Despite this, the differences among all methods become negligible when the sample size increases. For case III, the PMS generally outperformed the other methods in estimating the shape parameter *c*, as it achieved the lowest RMSE values across most sample sizes. On the other hand, both LS and CVM methods exhibited similar performance, for all three cases, as they generally produced slightly larger RMSE values than the ML and MPS methods, particularly for small sample sizes. Overall, the results demonstrated that all estimation techniques are capable of producing accurate parameter estimates, although the MPS method exhibits a slight advantage, particularly for small and moderate sample sizes.

## 7. Real Application

To investigate the applicability of the CAO–W distribution in fitting real data, four real datasets with various hazard behavior are considered. The CAO–W model is fitted to each dataset and its flexibility is compared to a set of competing distributions which are known in the literature for their flexibility in modeling various forms of lifetime data. These distributions are listed in Table 5.

In order to evaluate the flexibility of the proposed CAO–W distribution compared to the distributions outlined above, a set of information criteria is used namely Akaike Information Criterion (AIC), Bayesian Information Criterion (BIC), Hannan–Quinn Information Criterion (HQIC), Corrected Akaike Information Criterion (AICc), and Consistent Akaike Information Criterion (CAIC), as well as goodness-of-fit measures namely the Kolmogorov–Smirnov (KS) test and the corresponding *p*-value. Additionally, to provide a comprehensive assessment of the distribution performance, a graphical assessment is conducted using the PDF, the Kaplan–Meier (KM) estimates of the survival function and the cumulative hazard function.

### 7.1. Data Description

**Dataset 1**

This dataset, presented in [31], represents the time-to-failure records of 40 turbocharger suits which are essential components in the turbo-charged diesel engine. The observed values are

$$\begin{array}{c} 1.6,2.0,2.6,3.0,3.5,3.9,4.5,4.6,4.8,5.0,5.1,5.3,5.4,5.6,5.8,6.0,6.0,6.1,6.3,6.5,6.5,6.7,7.0,\\ 7.1,7.3,7.3,7.3,7.7,7.7,7.8,7.9,8.0,8.1,8.3,8.4,8.4,8.5,8.7,8.8,9.0. \end{array}$$

**Figure 7:**
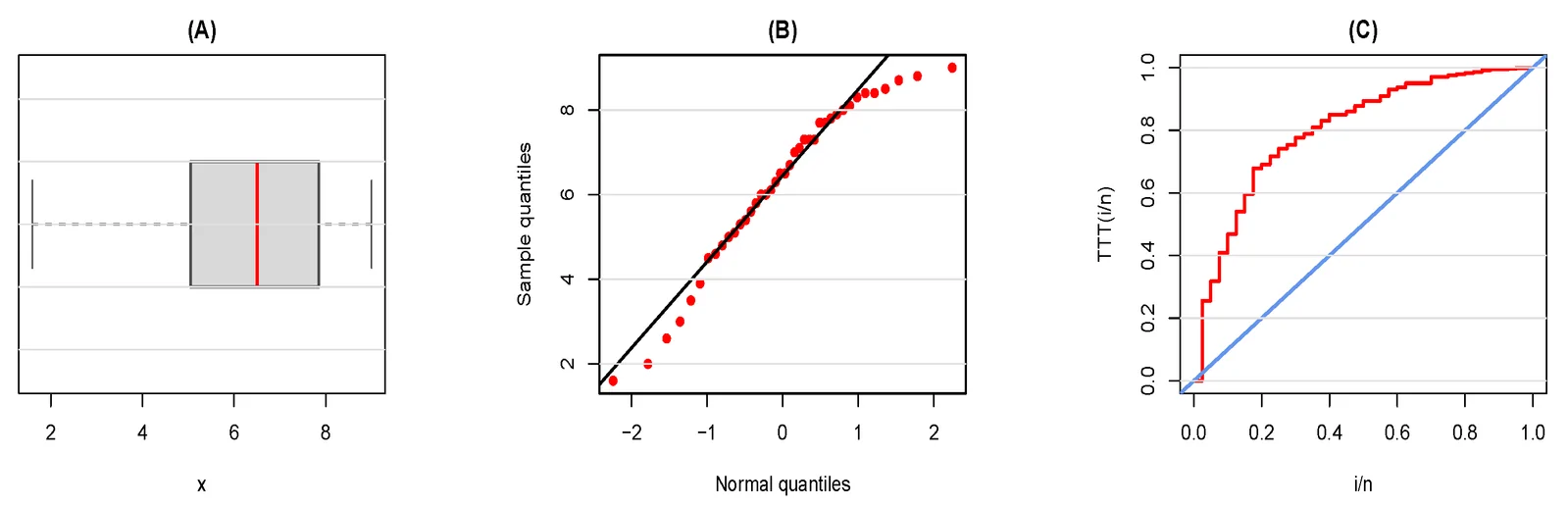
Descriptive of Dataset 1: (**A**): boxplot, (**B**): Q-Q plot, and (**C**): TTT plot.

Figure 7 describes the data graphically, whereas Table 6 provides a summary using some statistical measures.

As can be seen from the Q-Q plot and TTT plot, there is a departure from normality with a decreasing hazard behavior. Moreover, the table shows that the data has moderate variability with a slight negative skewness.

**Dataset 2**

This dataset provides time between failures of secondary reactor pumps. The dataset was originally presented [32] and used by [33]. The observations are given as follows:

$$\begin{array}{c} 2.160,0.150,4.082,0.746,0.358,0.199,0.402,0.101,0.605,0.954,1.359,0.273,0.491,3.465,\\ 0.070,6.560,1.060,0.062,4.992,0.614,5.320,0.347,1.921 \end{array}$$

**Figure 8:**
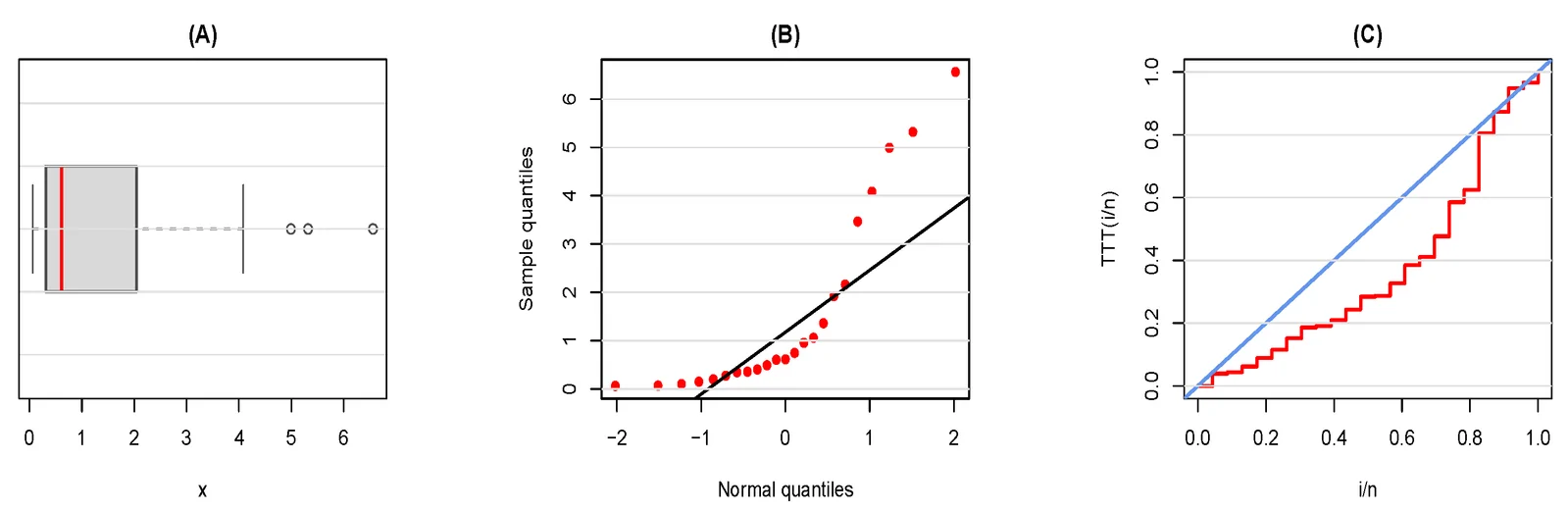
Descriptive of Dataset 2: (**A**): boxplot, (**B**): Q-Q plot, and (**C**): TTT plot.

Figure 8 provides a visual representation of the data, while Table 7 provides a statistical summary of the data.

The above figure and table reveal that the dataset has a clear departure from normality, an increase hazard behavior, and a moderate dispersion with positive skewness, along with a moderately heavy tail distribution.

**Dataset 3**

This dataset contains information concerning a large system with 30 units, where failures and running times are recorded. The dataset was reported in [34] and used in [35,36]. The observations are

$$\begin{array}{c} 2.75,0.13,1.47,0.23,1.81,0.30,0.65,0.10,3.00,1.73,1.06,3.00,3.00,2.12,3.00,3.00,3.00,0.02,\\ 2.61,2.93,0.88,2.47,0.28,1.43,3.00,0.23,3.00,0.80,2.45,2.66 \end{array}$$

**Figure 9:**
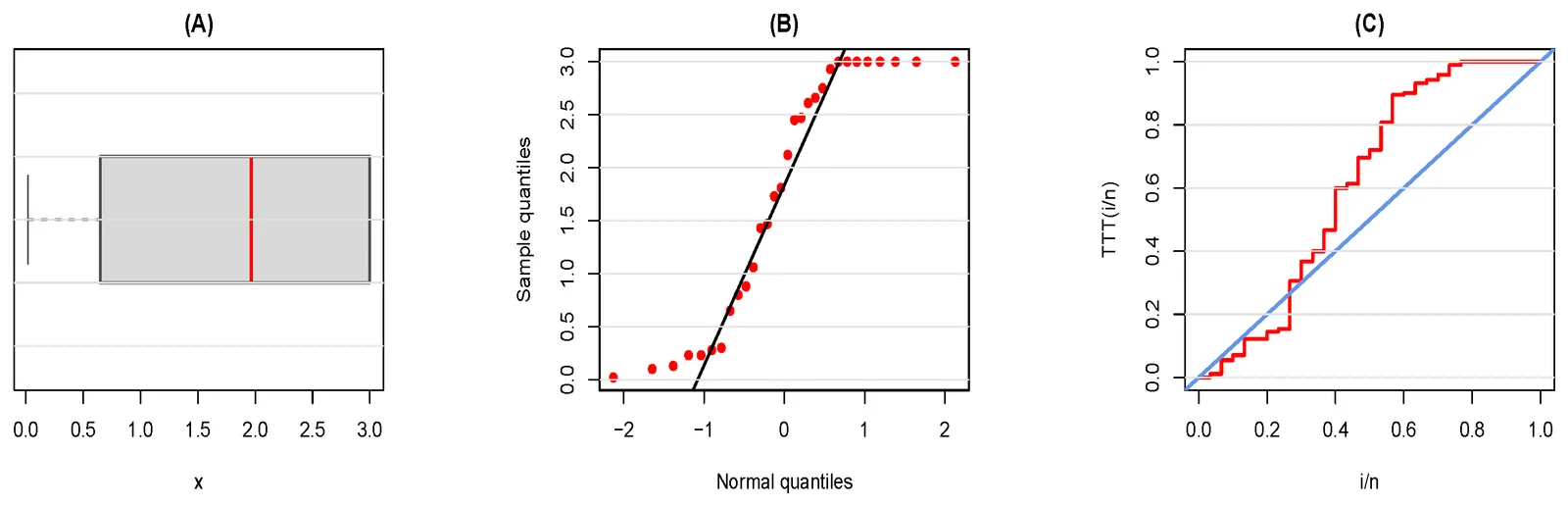
Descriptive of Dataset 3: (**A**): boxplot, (**B**): Q-Q plot, and (**C**): TTT plot.

A visual representation of the data is shown in Figure 9, while a summary using some statistical measures is given in Table 8.

It is evident from the figure and table above that the dataset has a non-normal distribution with an S-shaped hazard pattern, indicating a non-monotone hazard behavior. Furthermore, the dataset shows moderate dispersion and a slight negative skewness, as well as light tails.

**Dataset 4**

This dataset was discussed by [37], and corresponds to the period of relief from symptoms (in weeks) for a sample of 30 patients who suffered from leukemia and were treated similarly. The dataset was also used by [38] and the observations are as follows:

$$\begin{array}{c} 1,1,2,4,4,6,6,6,7,8,9,9,10,12,13,14,18,19,24,26,29,31,42,45,50,57,60,71,85,91. \end{array}$$

**Figure 10:**
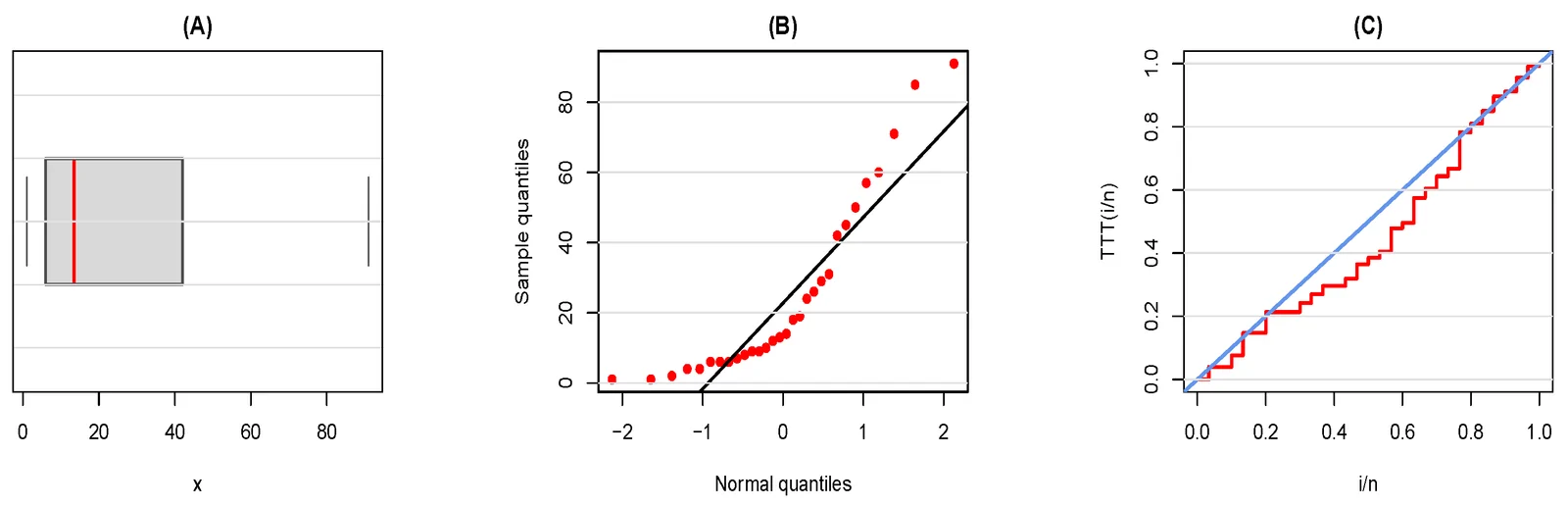
Descriptive of Dataset 4: (**A**): boxplot, (**B**): Q-Q plot, and (**C**): TTT plot.

Figure 10 illustrates the data graphically, while Table 9 provides some statistical measures.

It can be noted that the dataset has a non-normal distribution with mostly near constant, with a slight increasing, hazard behavior. Moreover, the dataset is highly variable and moderately positive skewed with a heavy upper tail.

### 7.2. Models Comparison

**Figure 11:**
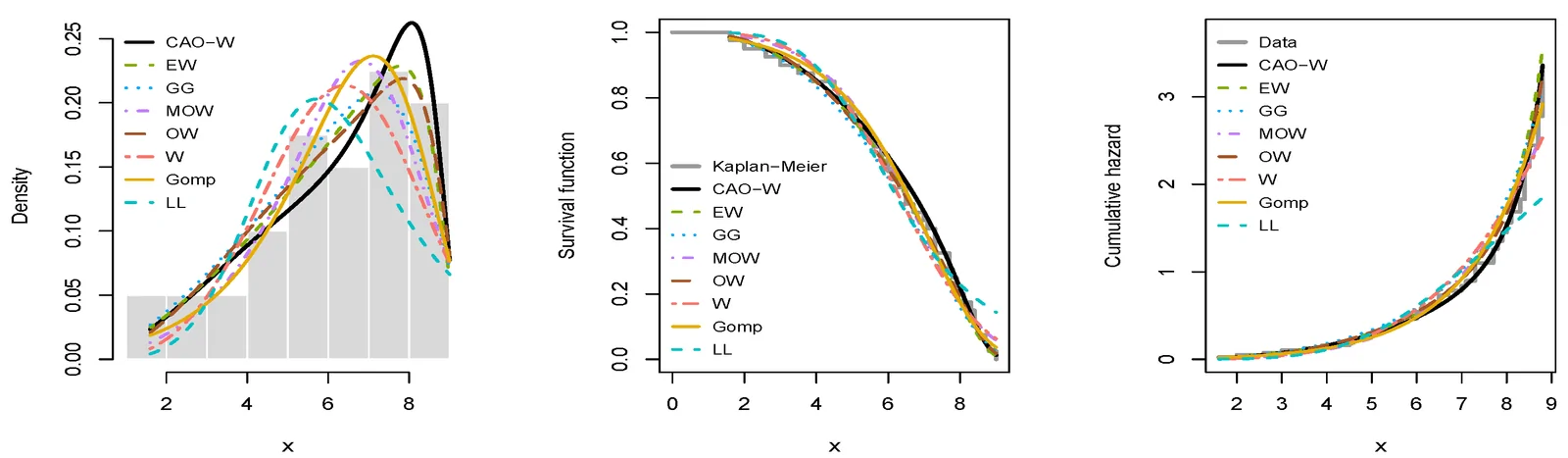
Graphical assessment for Dataset 1: (**left**): the empirical PDF, (**middle**): KM estimate and fitted curves, and (**right**): empirical and fitted cumulative hazard.

**Figure 12:**
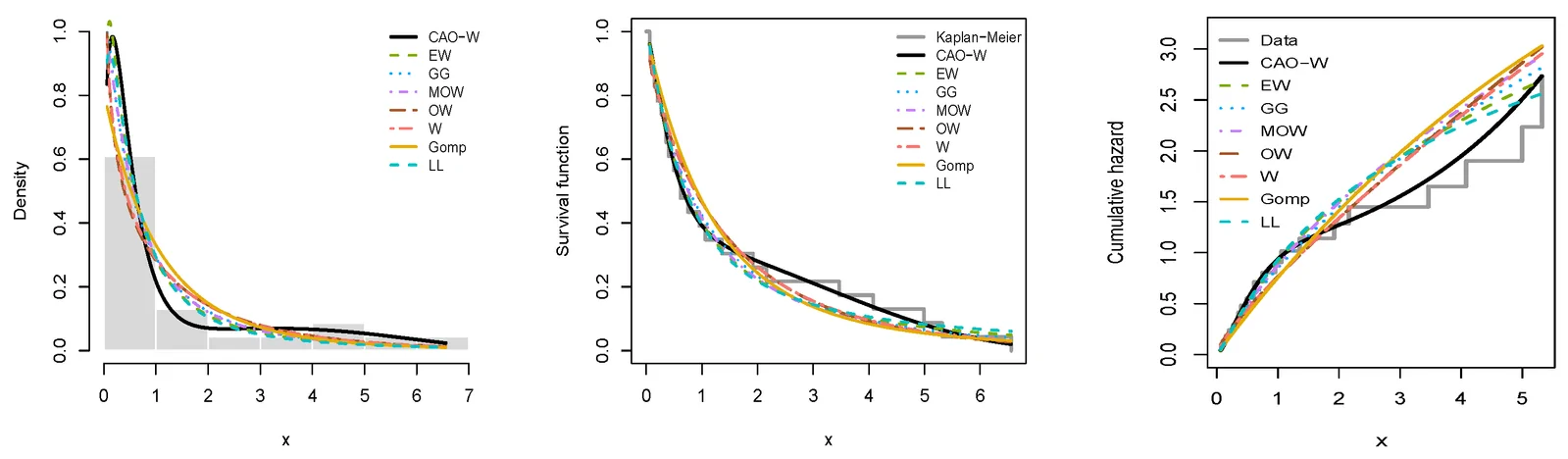
Graphical assessment for Dataset 2: (**left**): the empirical PDF, (**middle**): KM estimate and fitted curves, and (**right**): empirical and fitted cumulative hazard.

**Figure 13:**
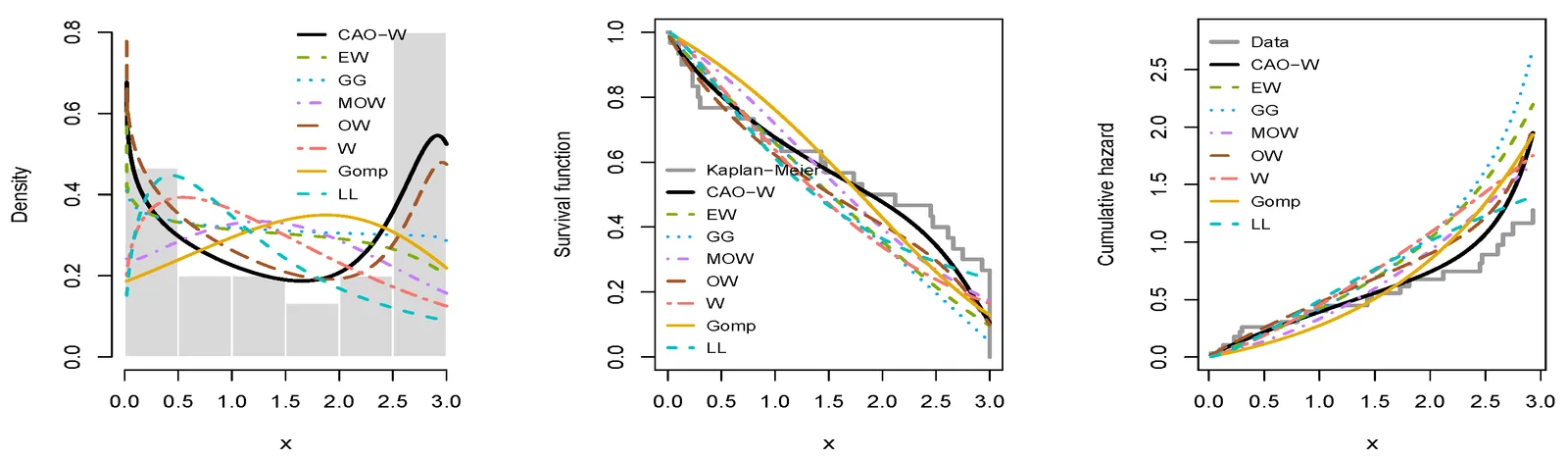
Graphical assessment for Dataset 3: (**left**): the empirical PDF, (**middle**): KM estimate and fitted curves, and (**right**): empirical and fitted cumulative hazard.

**Figure 14:**
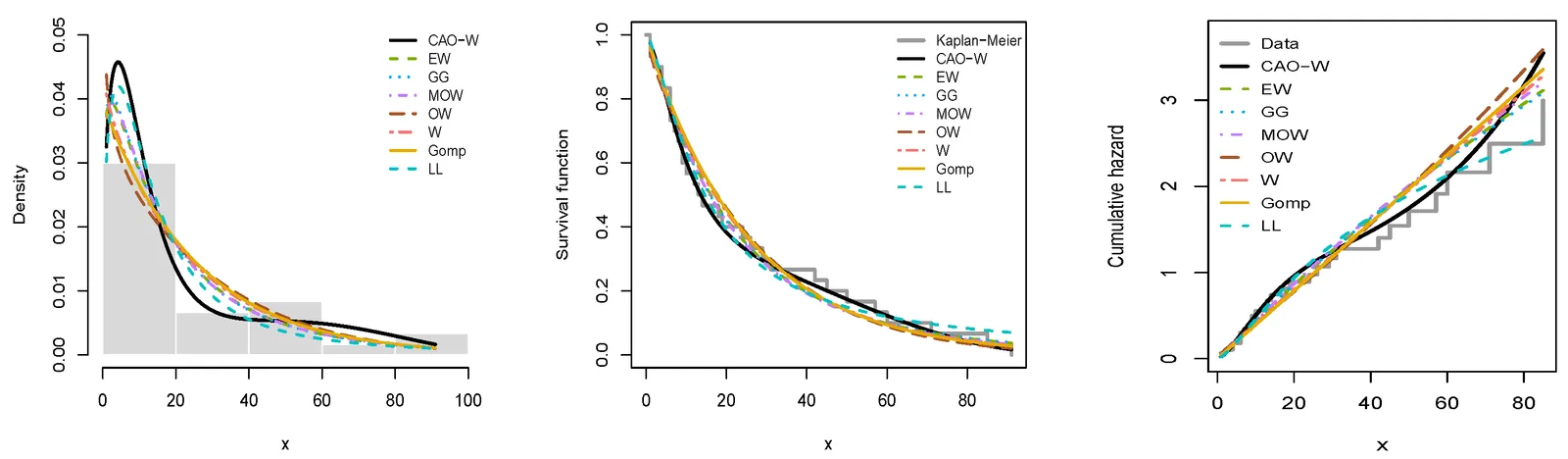
Graphical assessment for Dataset 4: (**left**): the empirical PDF, (**middle**): KM estimate and fitted curves, and (**right**): empirical and fitted cumulative hazard.

The performance of the CAO—W distribution is evaluated against the selected competing distributions through goodness-of-fit measures and graphical assessments. The results are summarized in Table 10, Table 11, Table 12 and Table 13, which report the maximum likelihood estimates (MLEs), standard errors (SEs), information criteria, and goodness-of-fit measures for each model, and presented in Figure 11, Figure 12, Figure 13 and Figure 14.

According to the Table 10, Table 11, Table 12 and Table 13 and Figure 11, Figure 12, Figure 13 and Figure 14, although all fitted models are consistent with the empirical behavior, the proposed distribution shows a stable and reliable fit. This observation is supported by the plot of the density, KM survival, and the cumulative hazard which indicate the greater flexibility of the CAO–W distribution in capturing the tail behavior, while preserving consistent fit across the entire range of the data.

### 7.3. Change Point Analysis

To further exemplify the proposed distribution, a single change-point analysis is conducted using the MIC. The purpose is to determine if it would be more accurate to represent the data by a single CAO–W distribution than two CAO–W distributions divided by an unknown change point. Let $x_{\left(1\right)},x_{\left(2\right)},...,x_{\left(n\right)},$ denote the ordered sample. For each candidate change point *k*, the data are divided into two parts, $x_{\left(1\right)},...,x_{\left(k\right)}$ and $x_{\left(k+1\right)},...,x_{\left(n\right)}$. The CAO–W distribution is individually fitted to each part, and the associated MIC value is calculated. Then, the estimated change point is obtained as follows

$$\hat{k}=\arg\min_{k}\left\{-2\log L_{1}\left({\hat{\Lambda }}_{1},{\hat{\Lambda }}_{2}\right)+\left[4+{\left(\frac{2k}{n}-1\right)}^{2}\right]\log n\right\},$$

where $L_{1}\left({\hat{\Lambda }}_{1},{\hat{\Lambda }}_{2}\right)$ indicates the likelihood obtained by fitting the CAO–W distribution independently to the observations before and after the candidate change point *k*. Table 14 presents the change point results derived using MIC for the four datasets, whereas Table 15 shows the parameter estimations before and after the identified change point for datasets exhibiting structural change.

**Figure 15:**
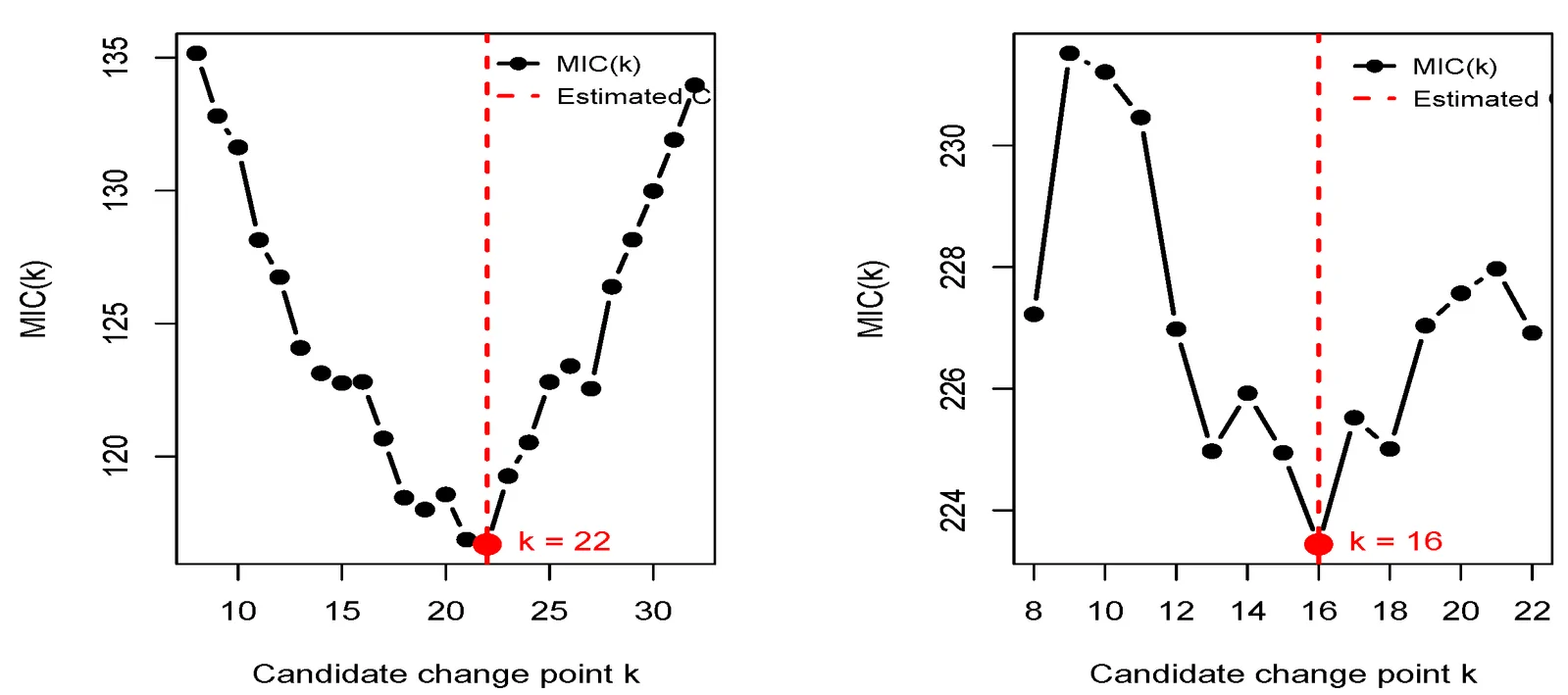
MIC curves for identifying the optimal change point in the dataset 1 (**left**) and dataset 4 (**right**).

According to Table 14 and Table 15, the results demonstrated structural change in Datasets 1 and 4; however, Datasets 2 and 3 showed no such evidence. In Dataset 1, the minimum MIC value was attained at $\hat{k}=22$, corresponding to a failure time of 6.7. This result indicates that the observations before and after this number exhibit different distributional characteristics, suggesting that the assumption of a single homogeneous population may be inadequate. A similar tendency was observed in Dataset 4, with the estimated change point detected at $\hat{k}=16$, signifying a remission length of 14 weeks. The parameter estimations for the two segments show considerable divergence, particularly with the size and asymmetry parameters, signifying a substantial change in the underlying data behavior. Moreover, the CAO–W model with a change point yielded lower MIC values compared to the model without a change point, showing that incorporating a change point improves the fit for these datasets (Figure 15).

## 8. Conclusions

This paper developed a CAO–W distribution as a flexible and applicable model to analyze lifetime and reliability data. This distribution is constructed by using an asymmetric-odd transformation allowing it to accommodate a wide range of hazard behaviors. Some useful statistical properties for the CAO–W were presented to strengthen the model’s mathematical foundation. Moreover, four widely applied estimation approaches were utilized to estimate the model parameters and their performance was assessed through three cases of simulation studies. The results showed that all four estimation methods performed satisfactorily, and estimation accuracy consistently improved with increasing sample size. This demonstrates the reliability of the proposed estimation procedures. In addition, the model’s efficiency was investigated by using four real datasets with different hazard patterns. The results demonstrated that the CAO–W distribution achieves strong competitive performance and often provides a good fit relative to well-known competitive models statistically and graphically. The MIC-based change point analysis further demonstrates the applicability of the proposed model in handling heterogeneous lifetime data. Thus, the proposed distribution can be considered an effective and valuable addition to current distributions for modeling lifetime and reliability data due to its high flexibility and adaptability in modeling different hazard behaviors. Despite its flexibility, the proposed CAO–W distribution has some limitations. Like any parametric lifetime model, its performance depends on how well the assumed distribution matches the data and may not be optimal for every application. Moreover, the present study does not consider regression structures or Bayesian inference. These limitations provide opportunities for future research, such as developing regression models, exploring Bayesian estimation methods, and extending the model to more complex survival settings.

## Figures and Tables

**Table 1:** Comparison of the proposed CAO–G family with some related generated families.

Family	Generator	Mechanism	Distinction from CAO–G
Exponentiated–G [27]	$G{\left(x\right)}^{\alpha }$	Power	Uses G(x).
Odd–G [10]	${\left(\frac{G(x)}{1-G(x)}\right)}^{\theta }$	Exponentiated odds	Symmetric odds transformation.
Marshall–Olkin–G [7]	$\frac{\alpha G(x)}{1-(1-\alpha )G(x)}$	Survival adjustment	Alters only the survival component.
Beta–G [8]	$I_{G(x)}\left(\alpha ,\beta \right)$	Beta CDF	Applies Beta-weighted transformation.
Kumaraswamy–G [9]	$1-{\left(1-G{\left(x\right)}^{a}\right)}^{b}$	Kumaraswamy CDF	Uses bounded power transformations.
CAO–G	$1-\exp\;\left\{-\frac{G{\left(x\right)}^{a}}{{\left(1-G\left(x\right)\right)}^{1-a}}\right\}$	Asymmetric hazard	Joint asymmetric use of G(x) and 1−G(x).

**Table 2:** Values of CV, SK, and K for some selected cases.

Case	β	*c*	*a*	CV	SK	K
1	0.7	1.0	0.1	1.5140	1.6540	5.1191
2	1.1	1.3	0.2	1.1737	1.1163	3.2987
3	1.8	2.0	0.3	0.8156	0.4985	2.1178
4	2.9	3.1	0.4	0.5437	0.0016	1.9450
5	3.1	3.2	0.5	0.4816	−0.0377	2.0619
6	3.8	3.9	0.6	0.3848	−0.1685	2.2348
7	4.4	4.4	0.7	0.3346	−0.1635	2.3371
8	5.1	5.1	0.8	0.2953	−0.0895	2.3772

**Table 3:** Effect of individual parameter on selected descriptive measures of the CAO–W distribution.

β	*c*	*a*	Mean	Variance	Skewness	Kurtosis	CV
3.00	3.10	0.40	2.0014	1.1839	0.0016	1.9450	0.5437
4.00	3.10	0.40	2.6685	2.1047	0.0016	1.9450	0.5437
5.00	3.10	0.40	3.3356	3.2886	0.0016	1.9450	0.5437
6.00	3.10	0.40	4.0027	4.7356	0.0016	1.9450	0.5437
2.50	1.00	0.40	1.4026	2.6501	1.3803	4.4167	1.1607
2.50	2.00	0.40	1.5099	1.2266	0.4424	2.1610	0.7335
2.50	3.00	0.40	1.6550	0.8476	0.0321	1.9424	0.5563
2.50	4.00	0.40	1.7697	0.6428	−0.2246	2.0483	0.4530
2.50	3.10	0.10	1.0270	0.9861	0.4394	1.6809	0.9670
2.50	3.10	0.30	1.4871	0.8565	0.0664	1.8179	0.6223
2.50	3.10	0.50	1.8339	0.8183	−0.0086	2.0544	0.4933
2.50	3.10	0.70	2.1733	0.9559	0.1282	2.2162	0.4499

**Table 4:** Estimates, RMSE, and convergence rate for ML, LS, MPS, and CVM methods across sample sizes for the three cases.

Case	*n*	Param	ML			LS			MPS			CVM		
			Est.	RMSE	CR (%)	Est.	RMSE	CR (%)	Est.	RMSE	CR (%)	Est.	RMSE	CR (%)
I	25	β	1.2142	0.5092	99.98	1.2493	0.4807	99.90	1.2788	0.4874	99.94	1.1992	0.4625	99.84
		*c*	2.1579	3.1282	99.98	1.8946	0.9296	99.90	1.7483	0.8433	99.94	2.0088	1.0482	99.84
		*a*	0.5975	0.2310	99.98	0.5765	0.2266	99.90	0.5750	0.2057	99.94	0.5910	0.2295	99.84
	50	β	1.1988	0.3573	100.00	1.2355	0.3676	100.00	1.2372	0.3358	100.00	1.2087	0.3598	100.00
		*c*	1.6861	0.4409	100.00	1.6877	0.4853	100.00	1.5684	0.3135	100.00	1.7324	0.5262	100.00
		*a*	0.6069	0.1566	100.00	0.5848	0.1729	100.00	0.5925	0.1399	100.00	0.5933	0.1742	100.00
	100	β	1.1899	0.2429	100.00	1.2208	0.2771	100.00	1.2086	0.2287	100.00	1.2068	0.2738	100.00
		*c*	1.5695	0.1749	100.00	1.5876	0.2527	100.00	1.5156	0.1469	100.00	1.6061	0.2611	100.00
		*a*	0.6100	0.1038	100.00	0.5924	0.1284	100.00	0.6029	0.0943	100.00	0.5971	0.1287	100.00
	200	β	1.1842	0.1594	100.00	1.2023	0.2023	100.00	1.1910	0.1513	100.00	1.1951	0.2012	100.00
		*c*	1.5277	0.1002	100.00	1.5362	0.1491	100.00	1.5019	0.0904	100.00	1.5449	0.1517	100.00
		*a*	0.6100	0.0680	100.00	0.5998	0.0929	100.00	0.6076	0.0632	100.00	0.6023	0.0930	100.00
	400	β	1.1885	0.0939	100.00	1.2020	0.1447	100.00	1.1898	0.0903	100.00	1.1983	0.1443	100.00
		*c*	1.5100	0.0555	100.00	1.5203	0.0991	100.00	1.4993	0.0520	100.00	1.5245	0.1001	100.00
		*a*	0.6070	0.0400	100.00	0.6000	0.0666	100.00	0.6068	0.0380	100.00	0.6013	0.0667	100.00
II	25	β	1.8199	0.2705	100.00	1.8555	0.2637	100.00	1.8692	0.2727	99.96	1.8354	0.2536	99.98
		*c*	6.1356	8.8047	100.00	5.4529	1.9294	100.00	5.1247	1.9580	99.96	5.8340	2.2621	99.98
		*a*	0.7641	0.2017	100.00	0.7308	0.2062	100.00	0.7314	0.1983	99.96	0.7428	0.2025	99.98
	50	β	1.8069	0.1874	100.00	1.8360	0.1917	100.00	1.8434	0.1884	100.00	1.8261	0.1871	100.00
		*c*	5.1590	1.3464	100.00	5.0141	0.9433	100.00	4.7709	0.7265	100.00	5.1764	1.0395	100.00
		*a*	0.7872	0.1283	100.00	0.7603	0.1438	100.00	0.7617	0.1290	100.00	0.7668	0.1415	100.00
	100	β	1.8032	0.1271	100.00	1.8205	0.1361	100.00	1.8279	0.1287	100.00	1.8156	0.1342	100.00
		*c*	4.8977	0.4547	100.00	4.8343	0.5306	100.00	4.6864	0.3812	100.00	4.9114	0.5670	100.00
		*a*	0.7947	0.0829	100.00	0.7792	0.0960	100.00	0.7770	0.0849	100.00	0.7826	0.0949	100.00
	200	β	1.7959	0.0871	100.00	1.8059	0.0932	100.00	1.8112	0.0873	100.00	1.8035	0.0925	100.00
		*c*	4.8079	0.3014	100.00	4.7643	0.3640	100.00	4.6863	0.2670	100.00	4.8024	0.3772	100.00
		*a*	0.8008	0.0559	100.00	0.7923	0.0631	100.00	0.7897	0.0567	100.00	0.7940	0.0627	100.00
	400	β	1.7982	0.0586	100.00	1.8038	0.0651	100.00	1.8070	0.0590	100.00	1.8026	0.0649	100.00
		*c*	4.7621	0.1960	100.00	4.7375	0.2496	100.00	4.6956	0.1785	100.00	4.7564	0.2548	100.00
		*a*	0.8006	0.0374	100.00	0.7961	0.0439	100.00	0.7942	0.0379	100.00	0.7970	0.0437	100.00
III	25	β	2.3961	0.4683	99.98	2.3881	0.4749	99.88	2.4319	0.4209	99.78	2.3308	0.4912	99.84
		*c*	4.5462	8.1509	99.98	3.8790	2.1922	99.88	3.5387	1.7749	99.78	4.0852	2.4462	99.84
		*a*	0.4346	0.2077	99.98	0.4350	0.2224	99.88	0.4309	0.1803	99.78	0.4493	0.2328	99.84
	50	β	2.4476	0.2826	100.00	2.4383	0.3403	100.00	2.4560	0.2602	100.00	2.4078	0.3489	99.96
		*c*	3.3606	0.8694	100.00	3.4823	1.3234	100.00	3.1701	0.6620	100.00	3.5500	1.3781	99.96
		*a*	0.4255	0.1249	100.00	0.4191	0.1635	100.00	0.4273	0.1135	100.00	0.4271	0.1684	99.96
	100	β	2.4735	0.1509	100.00	2.4642	0.2443	100.00	2.4726	0.1452	100.00	2.4485	0.2483	100.00
		*c*	3.1557	0.3408	100.00	3.2727	0.7797	100.00	3.0949	0.2937	100.00	3.2993	0.7930	100.00
		*a*	0.4155	0.0672	100.00	0.4120	0.1185	100.00	0.4178	0.0649	100.00	0.4161	0.1205	100.00
	200	β	2.4907	0.0660	100.00	2.4740	0.1755	100.00	2.4900	0.0652	100.00	2.4660	0.1774	100.00
		*c*	3.1070	0.1398	100.00	3.1583	0.5025	100.00	3.0945	0.1276	100.00	3.1700	0.5059	100.00
		*a*	0.4056	0.0303	100.00	0.4097	0.0848	100.00	0.4062	0.0305	100.00	0.4118	0.0857	100.00
	400	β	2.4989	0.0175	100.00	2.4869	0.1199	100.00	2.4990	0.0166	100.00	2.4829	0.1207	100.00
		*c*	3.1003	0.0330	100.00	3.1324	0.3466	100.00	3.0995	0.0291	100.00	3.1379	0.3478	100.00
		*a*	0.4007	0.0085	100.00	0.4053	0.0591	100.00	0.4007	0.0084	100.00	0.4063	0.0595	100.00

**Table 5:** Probability density functions of the competing distributions used in the real-data analysis.

Distribution	Probability Density Function
Weibull (W) [3]	$f\left(x\right)=\frac{c}{\beta }{\left(\frac{x}{\beta }\right)}^{c-1}\exp\;\left[-{\left(\frac{x}{\beta }\right)}^{c}\right],\;x>0,\;c,\beta >0.$
Exponentiated Weibull (EW) [29]	$f\left(x\right)=\alpha \frac{c}{\beta }{\left(\frac{x}{\beta }\right)}^{c-1}e^{-{\left(x/\beta \right)}^{c}}{\left(1-e^{-{\left(x/\beta \right)}^{c}}\right)}^{\alpha -1},\;x>0,\;\alpha ,\beta ,c>0.$
Gompertz (Gomp) [1]	$f\left(x\right)=be^{ax}\exp\;\left[-\frac{b}{a}\left(e^{ax}-1\right)\right],\;x>0,\;b>0,\;a\in R.$
Generalized Gamma (GG) [5]	$f\left(x\right)=\frac{c}{\Gamma \left(\alpha \right)\beta^{\alpha c}}x^{\alpha c-1}\exp\;\left[-{\left(\frac{x}{\beta }\right)}^{c}\right],\;x>0,\;\alpha ,\;\beta ,\;c>0.$
Log-Logistic (LL) [4]	$f\left(x\right)=\frac{k}{\alpha }{\left(\frac{x}{\alpha }\right)}^{k-1}{\left[1+{\left(\frac{x}{\alpha }\right)}^{k}\right]}^{-2},\;x>0,\;\alpha ,\;k>0.$
Marshall–Olkin Weibull (MOW) [7]	$f\left(x\right)=\frac{\alpha \frac{c}{\beta }{\left(\frac{x}{\beta }\right)}^{c-1}\exp\;\left[-{\left(\frac{x}{\beta }\right)}^{c}\right]}{{\left[1-\left(1-\alpha \right)\exp\;\left[-{\left(\frac{x}{\beta }\right)}^{c}\right]\right]}^{2}},\;x>0,\;\alpha ,\;\beta ,\;c>0.$
Odd Weibull (OW) [30]	$\begin{array}{c} f\left(x\right)=\alpha \frac{c}{\beta }{\left(\frac{x}{\beta }\right)}^{c-1}\exp\;\left[{\left(\frac{x}{\beta }\right)}^{c}\right]{\left[\exp\;\left({\left(\frac{x}{\beta }\right)}^{c}\right)-1\right]}^{\alpha -1}\\ \;\times \exp\;\left\{-{\left[\exp\;\left({\left(\frac{x}{\beta }\right)}^{c}\right)-1\right]}^{\alpha }\right\},\;x>0,\;\alpha ,\;\beta ,\;c>0. \end{array}$

**Table 6:** Descriptive statistics for Dataset 1.

Mean	SD	Min	Max	Skewness	Kurtosis
6.2525	1.9555	1.6000	9.0000	−0.6626	2.6410

**Table 7:** Descriptive statistics for Dataset 2.

Mean	SD	Min	Max	Skewness	Kurtosis
1.5778	1.9306	0.06200	6.5600	1.3643	3.5445

**Table 8:** Descriptive statistics for Dataset 3.

Mean	SD	Min	Max	Skewness	Kurtosis
1.7703	1.1499	0.0200	3.0000	−0.2840	1.4537

**Table 9:** Descriptive statistics for Dataset 4.

Mean	SD	Min	Max	Skewness	Kurtosis
25.3333	25.7311	1.0000	91.0000	1.1920	3.3341

**Table 10:** MLEs (SEs) with information criteria and goodness-of-fit measures for all models in Dataset 1.

Model	Parameter Estimates: MLE (SE)			Information Criteria					KS (*p*-Value)
				AIC	BIC	HQIC	AICc	CAIC	
CAO–W (β,c,a)	8.0213 (0.3957)	7.1177 (1.8407)	0.3613 (0.1244	161.9701	167.0367	163.8020	162.6368	167.0367	0.0537 (0.9998)
EW (β,c,α)	8.6133 (0.0074)	20.3236 (0.0074)	0.1207 (0.0190)	162.0445	167.1111	163.8764	162.7112	167.1111	0.0799 (0.9602)
GG (β,k,c)	8.6644 (0.3062)	0.2220 (0.0746)	10.9968 (3.1335)	164.5734	169.6400	166.4053	165.2400	169.6400	0.1085 (0.7337)
MOW (β,c,α)	5.2984 (1.3124)	2.7859 (0.8714)	4.8657 (5.6664)	168.6406	173.7072	170.4725	169.3073	173.7072	0.0917 (0.8888)
OW (β,c,α)	7.5451 (0.2275)	6.9947 (3.1217)	0.4091 (0.2040)	162.2700	167.3366	164.1019	162.9366	167.3366	0.0714 (0.9868)
W (β,c)	6.9200 (0.2947)	3.8722 (0.5175)	—	168.9510	172.3287	170.1723	169.2753	172.3287	0.1076 (0.7424)
Gomp (b,a)	0.0069 (0.0031)	0.6360 (0.0723)	—	163.8956	167.2734	165.1169	164.2199	167.2734	0.0888 (0.9101)
LL (k,α)	4.8413 (0.6536)	6.2251 (0.3480)	—	181.4132	184.7910	182.6345	181.7375	184.7910	0.1437 (0.3803)

**Table 11:** MLEs (SEs) with information criteria and goodness-of-fit measures for all models in Dataset 2.

Model	Parameter Estimates: MLE (SE)			Information Criteria					KS (*p*-Value)
				AIC	BIC	HQIC	AICc	CAIC	
CAO–W (β,c,a)	0.6433 (0.1932	1.4394 (0.2775)	0.9519 (0.0400)	65.3229	68.7293	66.1796	66.5860	68.7293	0.0565 (0.9999)
EW (β,c,α)	0.0241 (0.0814)	0.2986 (0.1420)	10.4439 (15.2401)	69.6639	73.0704	70.5206	70.9270	73.0704	0.0969 (0.9677)
GG (β,k,c)	0.0088 (0.0113)	4.8612 (1.2986)	0.3378 (0.0509)	69.8398	73.2463	70.6965	71.1030	73.2463	0.0979 (0.9647)
MOW (β,c,α)	3.6043 (3.1153)	1.0822 (0.2621)	0.2018 (0.2972)	69.8050	73.2115	70.6617	71.0682	73.2115	0.1058 (0.9351)
OW (β,c,α)	26.5976 (34.1764)	0.1243 (0.0550)	4.6674 (2.1481)	71.5012	74.9077	72.3579	72.7643	74.9077	0.1156 (0.8837)
W (β,c)	1.3913 (0.3804)	0.8076 (0.1297)	—	69.0278	71.2988	69.5989	69.6278	71.2988	0.1183 (0.8670)
Gomp (b,a)	0.8108 (0.2479)	−0.1414 (0.1417)	—	69.9077	72.1787	70.4789	70.5077	72.1787	0.1424 (0.6867)
LL (k,α)	1.2292 (0.2095)	0.7066 (0.2122)	—	69.2273	71.4983	69.7984	69.8273	71.4983	0.0933 (0.9770)

**Table 12:** MLEs (SEs) with information criteria and goodness-of-fit measures for all models in Dataset 3.

Model	Parameter Estimates: MLE (SE)			Information Criteria					KS (*p*-Value)
				AIC	BIC	HQIC	AICc	CAIC	
CAO–W (β,c,a)	3.0048 (0.0039)	7.0794 (0.0039)	0.1201 (0.0261)	66.4551	70.6587	67.7999	67.3782	70.6587	0.1619 (0.4106)
EW (β,c,α)	3.2180 (0.0025)	7.1496 (0.0025)	0.1286 (0.0234)	82.8922	87.0958	84.2369	83.8152	87.0958	0.2378 (0.0671)
GG (β,k,c)	3.1915 (0.1489)	0.0178 (0.0121)	52.3429 (36.1484)	75.5544	79.7580	76.8992	76.4775	79.7580	0.2552 (0.0401)
MOW (β,c,α)	0.8589 (0.5183)	0.9564 (0.2666)	5.5281 (5.6513)	95.1928	99.3963	96.5375	96.1158	99.3963	0.1872 (0.2433)
OW (β,c,α)	2.3091 (0.0033)	7.3969 (0.0033)	0.1204 (0.0171)	67.0569	71.2605	68.4017	67.9800	71.2605	0.1667 (0.3743)
W (β,c)	1.8803 (0.2821)	1.2649 (0.2044)	—	96.3174	99.1198	97.2139	96.7619	99.1198	0.2194 (0.1111)
Gomp (b,a)	0.1846 (0.0802)	0.7403 (0.2227)	—	86.6919	89.4942	87.5884	87.1363	89.4942	0.1888 (0.2348)
LL (k,α)	1.4607 (0.2274)	1.3662 (0.2969)	—	107.8424	110.6448	108.7389	108.2869	110.6448	0.2406 (0.0618)

**Table 13:** MLEs (SEs) with information criteria and goodness-of-fit measures for all models in Dataset 4.

Model	Parameter Estimates: MLE (SE)			Information Criteria					KS (*p*-Value)
				AIC	BIC	HQIC	AICc	CAIC	
CAO–W (β,c,a)	13.9547 (3.9691)	1.5391 (0.2586)	0.9215 (0.0563)	255.8961	260.0997	257.2408	256.8192	260.0997	0.0721 (0.9976)
EW (β,c,α)	13.1365 (22.4934)	0.7017 (0.5025)	1.8282 (2.5531)	259.6671	263.8707	261.0119	260.5902	263.8707	0.0845 (0.9828)
GG (β,k,c)	4.1048 (12.5611)	2.4850 (2.6903)	0.5699 (0.3494)	259.6018	263.8054	260.9466	260.5249	263.8054	0.0849 (0.9820)
MOW (β,c,α)	41.4449 (26.7895)	1.1684 (0.2861)	0.3836 (0.4834)	259.3391	263.5426	260.6838	260.2621	263.5426	0.0877 (0.9748)
OW (β,c,α)	71.7823 (53.7683)	0.3580 (0.2808)	2.0028 (1.5901)	260.5849	264.7885	261.9297	261.5080	264.7885	0.1074 (0.8793)
W (β,c)	25.0022 (4.9643)	0.9714 (0.1388)	—	257.8854	260.6878	258.7819	258.3298	260.6878	0.0992 (0.9290)
Gomp (b,a)	0.0393 (0.0110)	0.0000 (0.0084)	—	257.9271	260.7295	258.8236	258.3716	260.7295	0.1092 (0.8662)
LL (k,α)	1.4172 (0.2124)	14.6522 (3.3246)	—	260.1505	262.9529	261.0470	260.5949	262.9529	0.0831 (0.9856)

**Table 14:** Change point results derived using MIC for the four datasets.

Dataset	*n*	MIC(n)	minMIC(k)	$\hat{k}$	Evidence of CP
Dataset 1	40	163.2242	116.6939	22	Yes
Dataset 2	23	65.5939	65.9275	15	No
Dataset 3	30	65.8523	67.0815	8	No
Dataset 4	30	256.6985	223.4400	16	Yes

**Table 15:** Parameter estimations before and after the identified change point for datasets exhibiting structural change.

Dataset	$\hat{k}$	CP Value	Before CP			After CP		
			${\hat{\beta }}_{1}$	${\hat{c}}_{1}$	${\hat{a}}_{1}$	${\hat{\beta }}_{2}$	${\hat{c}}_{2}$	${\hat{a}}_{2}$
Dataset 1	22	6.7	6.2949	10.2736	0.2536	7.9940	21.9125	0.8946
Dataset 4	16	14.0	10.1009	2.7827	0.4693	41.4353	3.3248	0.9089

## Data Availability

Data is contained within the article.
